# Transport Model of Underground Sediment in Soils

**DOI:** 10.1155/2013/367918

**Published:** 2013-10-28

**Authors:** Sun Jichao, Wang Guangqian

**Affiliations:** ^1^School of Water Resources and Environment, China University of Geosciences, Beijing 100083, China; ^2^State Key Laboratory of Hydroscience and Engineering, Tsinghua University, Beijing 100084, China

## Abstract

Studies about sediment erosion were mainly concentrated on the river channel sediment, the terrestrial sediment, and the underground sediment. The transport process of underground sediment is studied in the paper. The concept of the flush potential sediment is founded. The transport equation with stable saturated seepage is set up, and the relations between the flush potential sediment and water sediment are discussed. Flushing of underground sediment begins with small particles, and large particles will be taken away later. The pore ratio of the soil increases gradually. The flow ultimately becomes direct water seepage, and the sediment concentration at the same position in the water decreases over time. The concentration of maximal flushing potential sediment decreases along the path. The underground sediment flushing model reflects the flushing mechanism of underground sediment.

## 1. Introduction

**Figure 1:**
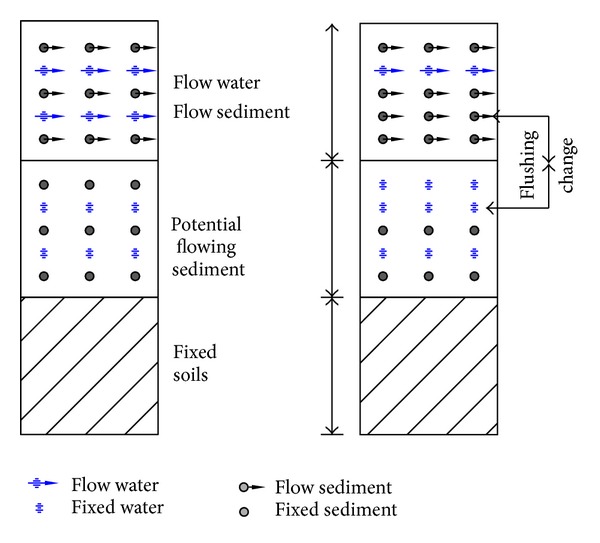
Schematics of particle exchange and transport of underground sediment.

The transport of the sediment in the soil driven by underground water force is known as underground sediment transport, which is shown in Figure 1.

Previous studies about sediment were mainly concentrated on the river sediment, marine sediment, and terrestrial sediment. However, these studies focused on sediment on the surface generally [1]. A new research direction is the underground sediment [2, 3], the sediment inside the soil, and the new study enhances the level of research from the existing surface sediment to underground sediment.

The existence and transport of underground sediment will cause a lot of engineering problems. Sediment transport within the soil of the lower river course of changing water level results in sand draining and may eventually lead to the collapse of the dam embankment; changes of groundwater level could lead to surface collapse caused by underground sediment transport; sediment in faults and fissures is transported driven by external disturbances (such as earthquakes and water level fluctuations) and leads to the activation of faults and fractures; underground sediment is also transported with the movement of water and oil transport, causes blockage of oil fissures, and decreases the speed of oil production. All these problems may lead to major accidents or increase costs, and they are related with underground sediment transport directly.

## 2. Research Statuses

Research of the underground sediment transport involves in sediment movement mechanics and is interdisciplinary. There has been a lot of research on these two aspects.

Previous studies on sediment movement mechanics could be roughly divided into two areas: the mechanism of physical phenomena and engineering applications. There has been an extensive research on the first area which relates to settling, starting, moving, and bedload movement of sediment particles, the sediment carrying capacity, nonequilibrium sediment transport, gravity flow, river simulation, reservoir sedimentation, river evolution, and so forth [4]. 

These studies of the mechanism of physical phenomenon mechanism and engineering applications of sediment movement mechanics concentrate on sediment start, transport, and saturation, and their methods are indoor and outdoor experiments and mechanical analysis, carrying on sediment of the coast, river, river basin, and surface. These research papers have not been involved in underground sediment [2, 3].

Studies of permeation fluid mechanics in this area focus on the seepage of flow in saturated [5, 6] and unsaturated soils [7, 8]. Accompanied by the study of sediment transport, defining of the state point is conducted based on the experimental and numerical computation [9–12]. There are only a few studies on related theories of sediment transport in groundwater, especially in terms of the quantitative transport process.

The two processes of subsurface erosion and volume uplift [13] have been observed in the experiment of the two processes, with the conclusion that the irreversible displacement of each soil particle corresponds to a critical hydraulic gradient [14]. These results of the experiments have some practical values, but they lack necessary and sufficient proof of relevant theory. Therefore, they are not so rational and applicable in practical application.

Force analysis of particles in the pore channels is conducted in the literature [15], obtaining the critical flow velocities formula when sediment particles start to move. However, sediment particles are nonsticky sediment with the same diameter, which might be inconsistent with the actual situation as actual sediment. Thus, its application has certain limitations.

## 3. Underground Sediment Transport Equations with Stable Saturated Seepage

The assumption in the paper is that the diameter of underground sediment particles which can be transported is much smaller than the diameter of the rest of the soil particles.

**Figure 2:**
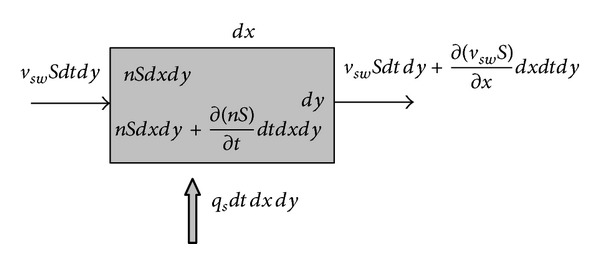
Unit body of underground sediment transport.

A small unit along the path of the underground sediment transport is studied, which is shown in Figure 2.

### 3.1. The Sediment Transport Equation

Within unit body *dxdy* and time *dt*, the sediment volume carried by the flow

(1) $$\begin{array}{c} \frac{\partial \left(v_{sw}S\right)}{\partial x}dxdtdy. \end{array}$$

Within unit body *dxdy* and time *dt*, the sediment volume in water increases as follows:

(2) $$\begin{array}{c} \frac{\partial \left(nS\right)}{\partial t}dtdxdy. \end{array}$$

Sediment in the *y* direction has no concentration gradient namely, there is no sediment diffusion in this direction; the sediment volume in water supplied by flush potential sediment is

(3) $$\begin{array}{c} q_{s}dtdxdy, \end{array}$$

wherein *q*_*s*_ is the volume of sediment in the soil entering the water per unit time per unit volume.

Therefore, within the unit body *dxdy* and time *dt*, the increased sediment equation is

(4) $$\begin{array}{c} \frac{\partial \left(nS\right)}{\partial t}dtdxdy=-\frac{\partial \left(v_{sw}S\right)}{\partial x}dxdtdy+q_{s}dtdxdy. \end{array}$$

The following equation is obtained after simplification:

(5) $$\begin{array}{c} \frac{\partial \left(nS\right)}{\partial t}=-\frac{\partial \left(v_{sw}S\right)}{\partial x}+q_{s}. \end{array}$$

### 3.2. Relations between the Flush Potential Sediment and Water Sediment

The sediment in water supplied by flush potential sediment is shown in Figure 2, and the volume is

(6) $$\begin{array}{c} q_{s}dtdxdy, \end{array}$$

wherein when *q*_*s*_ is positive, it indicates the sediment volume in soil entering in the water per unit time per unit volume; when it is negative, it indicates the speed of the sediment in cementing to the soil, which can be called the solventing speed.

The sediment will start to flow into the seepage water. As they are saturated underground sediments, the flow rate is constant. But at the same time, a part of the fixed sediment flows into the seepage water, with the end result that exchange happens between this part of sediment and flow water of the same volume; namely, the fixed sediment changes into flow sediment, and the flow water changes into fixed water of the same volume.

A new parameter, the flush pore ratio of the fixed sediment *n*_1_, is then introduced which meets

(7) $$\begin{array}{c} \frac{\partial n_{1}}{\partial t}=q_{s}, \end{array}$$

wherein *n*_1_ is the volume potential flush sediment changing from resting to moving, and the overall volume of the potential flush sediment is *n*_1max⁡_. Consider

(8) $$\begin{array}{c} n_{1\max}=n_{1}\left(t\to +\infty \right). \end{array}$$

The entry speed of potential sediment is directional; that is, on the one hand, the flush potential sediment in the soil flows into the seepage, and on the other hand, sediment in the seepage cements into the soil.

**Figure 3:**
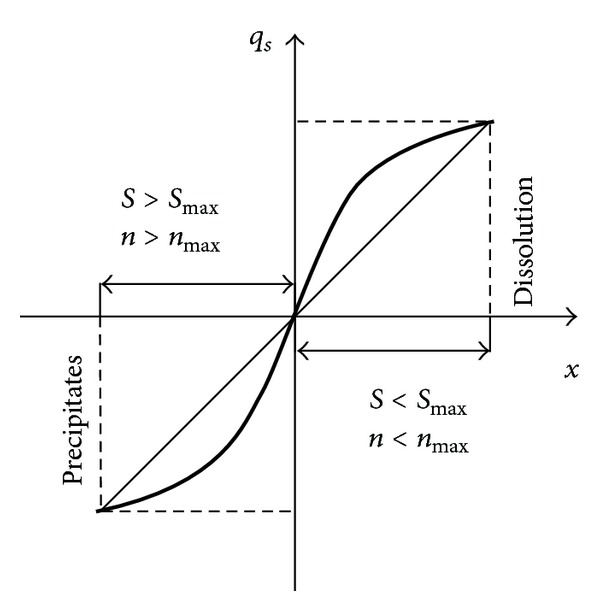
Relationship between solventing speed and greatest sediment concentration.

The speed is related to the particle size distribution, particle composition, the speed of seepage water, and seepage water sediment concentration of sediment in the soil. The specific relationship is as follows: the higher the seepage speed, the bigger the *q*_*s*_; the greater the seepage water sediment concentration, the smaller the *q*_*s*_; the greater the potential flush force of the sediment, the bigger the *q*_*s*_. The graphical representation is as in Figure 3.

The solventing speed *q*_*s*_ shown in Figure 3 complies with the relevant laws as follows:

(9) $$\begin{array}{c} q_{s}=K{\left(S_{\max}-S\right)}^{\alpha }, \end{array}$$

wherein *K* is the Solventing speed factor; *α* is the coefficient, as shown in Figure 3, taking 1, 3, 5,… and so forth, the odd function; *S*_max⁡  _ is the maximum seepage water sediment concentration, which can be called the greatest potential sediment concentration or potential sediment concentration for short. *S* is the seepage water sediment concentration.

The flushing process must satisfy the conditions of the pore ratio, which are as follows.

Dissolution: *S* < *S*_max⁡_, and it must satisfy *n*_1_ < *n*_1max⁡_. Cementing: *S* > *S*_max⁡_, and it must satisfy *n*_1_ > 0. The opposite situation is as follows, when *S* < *S*_max⁡_, but *n*_1_ ≥ *n*_1max⁡_, there is no dissolution, but *S* flush is maintained; when *S* > *S*_max⁡_, but *n*_1_ = 0 or *n*_1_ = *n*_1min⁡_, there is no cementing, and *S* flush is maintained. This could not happen because it refers to the fact that blocking occurs on the soil and the seepage has stopped.

The maximum sediment concentration of the seepage water *S*_max⁡_ is directly related to the speed of the soil seepage water. The smaller the seepage speed of the water, the smaller the *S*_max⁡_. There is an exponential relationship between the two, and relation [16] between the sediment concentration and flow rate is cubic; namely,

(10) $$\begin{array}{c} S_{\max}=K_{s}v_{sw}^{3}. \end{array}$$

The surface of the sediment particles is affected by the local vortex of low near the microcontact surface of the soil with water thus is carried away by the flow. Though there is local variation of the flow speed, it is very low in terms of the overall seepage flow. Thus, based on the potential sand concentration of river sediment carrying force, the potential sediment concentration formula of the underground sediment transport is determined as follows:

(11) $$\begin{array}{c} S_{\max}=K_{s}v_{sw}, \end{array}$$

wherein *K*_*s*_ is the coefficient of the maximum amount of sand carrying sediment.

The maximum pore ratio *n*_max⁡_ is related to the seepage water speed and the sediment particles gradation distribution in the soil. In view of the complicated sediment particle distribution, it is assumed that the gradation distribution does not affect the underground sediment transport, so only the speed of the seepage water is taken into account.

Referring to formula (9), the maximum pore ratio is taken as

(12) $$\begin{array}{c} n_{1\max}=K_{n1}v_{sw}. \end{array}$$

In the actual calculation, it can also be considered that the maximum pore ratio is constant value.

According to the formulas (7) and (9), it is obtained that

(13) $$\begin{array}{c} \frac{\partial n_{1}}{\partial t}=K{\left(K_{s}v_{sw}-S\right)}^{\alpha }. \end{array}$$

Simultaneous of (5), (7), and (13) helps in the formation of the underground sediment transport equation. 

The above underground sediment transport is saturated sediment flushing; thus, the hydroscience speed *v*_*sw*_ remains unchanged; the pore ratio n is the effective porosity of the underground sediment delivery ratio, which means that the pore ratio *n* involved in the sediment transport is also unchanged; the above equation can be simplified as

(14) $$\begin{array}{c} n\frac{\partial S}{\partial t}=-v_{sw}\frac{\partial S}{\partial x}+K{\left(K_{s}v_{sw}-S\right)}^{\alpha }. \end{array}$$

When *dt* = *dx*/*v*_*sw*_, the above equation becomes

(15) $$\begin{array}{c} v_{sw}\left(n+1\right)\frac{\partial S}{\partial x}=K{\left(K_{s}v_{sw}-S\right)}^{\alpha }. \end{array}$$

Further convert formula (15) to

(16) $$\begin{array}{c} v_{sw}\left(n+1\right)\frac{dS}{dx}=K{\left(K_{s}v_{sw}-S\right)}^{\alpha }. \end{array}$$

This is the underground sediment transport equation, which is also underground sediment distribution equation along the path.

## 4. Equation Solving

In terms of (16) solving, difficulties differs largely variable value of the parameter *α*. Equation (9) is of an odd function graphics, with the value of *α* being 1, 3, 5, 7,…; thus, the analytical calculation results of (16) are

 when *α* ≠ 1,
  (17) S=−(vsw(n+1)K(α−1)(x+C1))1/(α−1)+Ksvsw
 when *α* = 1,
  (18) $$\begin{array}{c} S=K_{s}v_{sw}+e^{-(Kx/v_{sw}(n+1))}C1, \end{array}$$

wherein *C*1 is a constant, and its specific value needs to be determined by the boundary condition.

During the flushing, at the moment of time *t*, three soil pore ratios are relative to the overall volume of the soil (including volume of the flowing water, moving sand, the potential static water, the potential static sand, and the fixed sand). Among it, the volume ratio of the flowing water sediment is *n*; when the time is 0, the volume ratio of the flowing water sediment is the initial pore ratio of the soil, *n* = *n*_0_; the volume of water of the flowing water sediment, namely, the flowing water ratio, is *n* × (1 − *S*); the pore ratio of the potential flowing sediment, namely, the volume ratio of the potential flowing sediment *n*_1max⁡_, is

(19) n1max⁡=n1(t→+∝)=∫0+∝K(Ksvsw−S)αdt.

The overall ratio of the soil, according formula (13), as the flowing sand cannot support the external mechanical effect of the soil, is also counted as the overall pore volume as follows:

(20) nt=n0+n1(t)=n0+∫0tK(Ksvsw−S)αdt.

Under real-time condition,

(21) nt(i+1)=nt(i)+n1t(i+1)=nt(i)+∫t(i)t(i+1)K(Ksvsw−S)αdt.

The boundary conditions are:

When *x* = 0, *S* = 0, and therefore a constant term of *F* is *K*_*svsw*_.

When *α* ≠ 1,

(22) $$\begin{array}{c} C1=\frac{\left(n+1\right)K_{s}v_{sw}^{2}}{Ke^{\alpha \ln\left(K_{s}v_{sw}\right)}\left(\alpha -1\right)}. \end{array}$$

After simplification, it is

(23) $$\begin{array}{c} C1=\frac{\left(n+1\right)K_{s}v_{sw}^{2}}{K{\left(K_{s}v_{sw}\right)}^{\alpha }\left(\alpha -1\right)}. \end{array}$$

When *α* = 1,

(24) $$\begin{array}{c} C1=-K_{s}v_{sw}. \end{array}$$

## 5. The Termination Condition of Underground Sediment Flushing

Flushing of the underground sediment is not always going following this rule unrestrictedly. During the flushing, as the pore ratio increases, the sediment decreases and will be washed away by the flow eventually. If there is no collapse in the soil, the pore ratio of this process will remain the same, and underground sediment transport will be replaced by groundwater seepage. Variable factors and parameters of the process can be ascribed as the potential sediment concentration *S*_max⁡_ could not simply adopt the formula *S*_max⁡_ = *K*_*s*_ *v*_*sw*_^3^, but only complies with it at the beginning of the flushing and decreases later on. It is directly related with pore ratio, and the maximal ratio adopts the ratio *n*_*w*_ formed finally at this flowing speed. In actual calculation, the maximal pore ratio can be deemed as a constant.

Therefore, the potential sediment concentration *S*_max⁡_ during the flushing is amended in the following formula:

(25) $$\begin{array}{c} S_{\max}=\frac{K_{s}v_{sw}^{}}{n_{1\max}-n_{0}}\left(n_{1\max}-n_{w}\right). \end{array}$$

The initial pore ratio is *n*_*w*0_ = *n*_0_.

## 6. Calculation Examples

### 6.1. The Initial State

**Figure 4:**
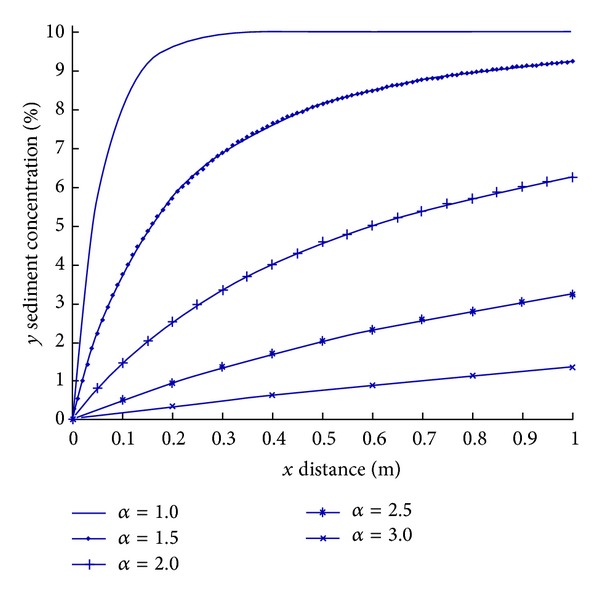
Sediment concentration distribution of initial transport along the path *x* (m), *y* sediment concentration (%).

The calculated parameters are as follows: *K*_*s*_ = 2, *v*_*sw*_ = 0.05 m/s, *K* = 1, *n* = 0.2, *x* = 0 → 1, and *n*_1max⁡_ = 0.6. According to (17) and (18), the sediment concentration is calculated with the results shown in Figure 4.

Furthermore, calculate the underground sediment process in accordance with (20) and (25). The calculation indicates that since the *K*_*s*_ *v*_*sw*_ of formula (16) is replaced by ((*K*_*s*_ *v*_*sw*_)/(*n*_1max⁡_ − *n*_0_))(*n*_1max⁡_ − *n*_*w*_) and also replaced according to (20), while (20) relates to the calculation of sediment content concentration *S*, the solution is to conduct iterative calculation in accordance with results of the initial state. The time variable *t* is introduced in (20) to obtain the results variation law during the flushing process of the underground sediment.

Equation (20) becomes

(26) nt(i+1)=nt(i)+∫t(i)t(i+1)K(Ksvswn1max⁡−n0(n1max⁡−nw)−S)αdt=nt(i)+[t(i+1)−t(i)]×K(Ksvswn1max⁡−n0(n1max⁡−nw)−S)α.

### 6.2. Model Calculations at *α* = 1

The study area has a length of 100 cm.

When *α* = 1 and within the time of 2000 seconds, the transport process of underground sediment is calculated.

From Figure 4, the following laws can be obtained.

1. The sediment concentration along the path increases with the increase of groundwater entrance distance, and the degree of its increase reduces with the distance to the entrance; the sediment concentration ultimately reaches a stable value.
2. The sediment concentration decreases with the increase of the *α* value.
3. During the initial period, the sediment concentration along the path decreases with the increase of the *α* value.

**Figure 5:**
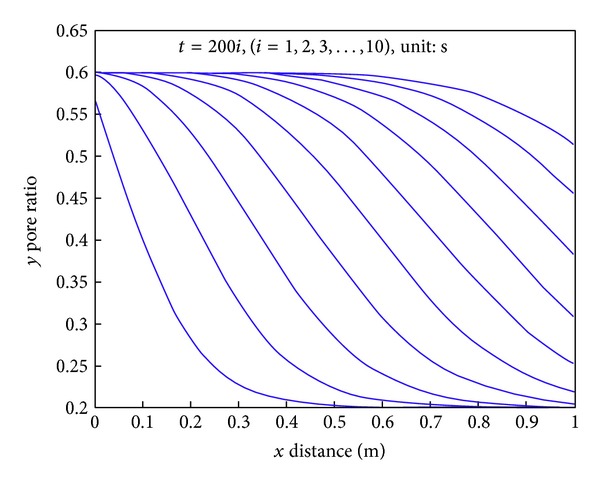
Changing of pore ratio along the path over time *x* (m), *y* pore ratio.

The following laws are obtained from Figure 5.

1. At the same moment, the pore ratio gradually reduces and finally approaches the minimum value.
2. At the same position, the pore ratio increase over time is increased and finally approaches the maximum value.
3. At the intermediate time, reducing the pore ratio along the path shows a law of increasing firstly and decreasing later on; in other words, the absolute value of the curve slope or that of the first derivative increases firstly and then decreases.

**Figure 6:**
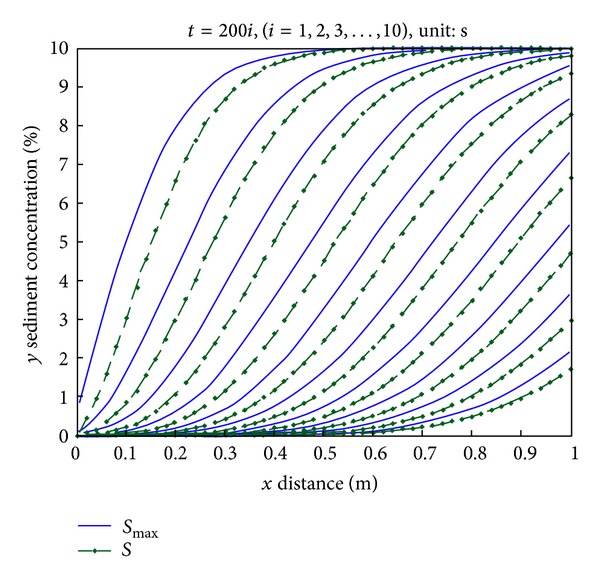
Changing of sediment concentration and potential sediment concentration along the path over time *x* (m), *y* sediment concentration (%).

The following laws are obtained from Figure 6.

1. Laws of the sediment concentration and potential sediment concentration show strong consistency and similarity. The potential sediment concentration of same location at the same time is greater than the sediment concentration.
2. At the same moment, the sediment (potential sediment concentration) concentration increases along the path gradually and eventually tends to be the potential sediment concentration (maximum potential sediment concentration).
3. Sediment concentration (potential sediment concentration) of the same position decreases over time and tends to be the minimum value eventually.
4. At the intermediate time, the increase of the sediment concentration (the potential sediment concentration) increases firstly and then decreases along the path; in other words, it shows a law of first increase and then decrease of the curve of the slope or first derivative.

### 6.3. Mechanistic Explanation

When the groundwater flows through the soil, static sediment along the path enters into the water and becomes flowing sediment; during the entire distance of the water course, static sediment could enter the water at any time; thus, the longer the flow path, the more the sediment in the water and vice versa.

Along with the flow path, the sediment concentration in the water increases, and it is also more difficult for static sediment to enter into the water. Therefore, the net difference between the sediment entering the water and the original sediment in the water which becomes static sediment later on gradually decreases, which means that the quality difference of the sediment entering into the water and sediment cementing in the water decreases. From a macropoint of view, the quality of static sediment entering the water is reducing and closing to zero; thus, the increase of sediment concentration is decreasing along the path.

A greater *α* value indicates that groundwater's flushing on sediment is stronger and more serious. At the very short period of the beginning, the quicker the water flows, the more the sediment is washed starting from this point. Therefore, the higher the flowing speed, the smaller the sediment concentration within the same distance along the path; namely, the bigger the *α* value, the smaller greater the sediment concentration under the same condition. Instead, the smaller the *α* value, sediment within the same distance along the path is less likely to be carried away, thus the greater the sediment concentration in the water.

With a certain distance from the entrance, the smaller the *α* value, the weaker the flushing force, so time spent on passing the same distance of both the flowing water and the flowing sediment is the same. With the period, more static sediments enter into the water. Therefore, in the initial period within the same distance, the sediment concentration increases more rapidly than the case of large values of *α*. In other words, in the initial period of distance, there is quick increase of the sediment concentration. In contrast, the larger the *α* value is, the slower the increasing degree of the sediment concentration is in the initial distance.

The groundwater seeps along the path and brings away the static sediment, changing it into flowing sediment, while the pore ratio at the same position increases; at the same moment, sediment along the path enters into the water gradually, which makes it difficult for sediment's entering later on; thus, the pore ratio of the following distance is small, showing a gradual decrease of the pore ratio along the distance at the same time.

Along the path, sediment enters into the water and becomes flowing sediment, so the sediment concentration of the water increases gradually.

At the same position, as some static sediments have become flowing sediments in the water, the total amount of sediments of such type is decreasing; as the soil contains many large and small particles, small particles are most likely to be taken away under the same flow, and large particles will be washed away when all possible small ones are taken. Since the remaining large particles could not be brought away, the sediment concentration is close to the minimum and tends to be 0, and the potential sediment concentration of this position also tends to 0.

## 7. Conclusions

Flushing of underground sediment begins with small particles, and large particles will be taken away later which is more difficult; the underground sediment flushing contains erosion and siltation, and the macroperformance between the two is their differences. When groundwater movement brings away underground sediment particles away, pore ratio of the soil increases gradually. When all possible particles are washed away with large particles left, the flow becomes direct water seepage ultimately, and the sediment concentration at the same position in the water decreases over time. The longer the path, the more the static sediment entering the water, and this reduces the increase of sediment concentration. High sediment concentration in the water will weaken static sediment's diffusion into the water, so the increase of the sediment concentration reduces; with a part of sediment entering into the water, the remaining part becomes less, so the concentration of maximal flushing potential sediment decreases along the path.

From the underground sediment flushing model, the above underground sediment flushing law is obtained, which reflects the flushing mechanism of underground sediment.

## References

[1] Lim WY, Aris AZ, Zakaria MP (2012). Spatial variability of metals in surface water and sediment in the langat river and geochemical factors that influence their water-sediment interactions. *Scientific World Journal*.

[2] Guangqian W, Jichao S, Xudong F, Jiahua W, Baosheng W An underground sand erosion model experimental apparatus and method.

[3] Guangqian W, Jichao S, Xudong F, Jiahua W, Baosheng W Underground sand erosion model experimental apparatus.

[4] Guangqian W (2007). Advances in river sediment research. *Journal of Sediment Research*.

[5] Jichao S, Guangqian W (2013). Research on underground water pollution caused by geological fault through radioactive stratum. *Journal of Radioanalytical and Nuclear Chemistry*.

[6] Sandor R, Fodor N (2012). Simulation of soil temperature dynamics with models using different concepts. *Scientific World Journal*.

[7] Sun J, Gao Q, Wang H, Li Y (2006). Numerical simulation of coupled rainfall and temperature of unsaturated soils. *Key Engineering Materials*.

[8] Sun J, Wang G, Sun Q (2009). Crack spacing of unsaturated soils in the critical state. *Chinese Science Bulletin*.

[9] Gabet EJ, Sternberg P (2008). The effects of vegetative ash on infiltration capacity, sediment transport, and the generation of progressively bulked debris flows. *Geomorphology*.

[10] Haynes H, Vignaga E, Holmes WM (2009). Using magnetic resonance imaging for experimental analysis of fine-sediment infiltration into gravel beds. *Sedimentology*.

[11] Roberge PR (2010). Polymeric materials for underground piping and related systems. *Materials Performance*.

[12] Fredlund DG, Rahardjo H (1993). *Soil Mechanics for Unsaturated Soils*.

[13] Terzaghi K (1922). Der grundbruch an stauwerken und seine verhuetung. *Die Wasserkraft*.

[14] Bazant Z Measuring soil deformation caused by the pressure of the seepage.

[15] Indraratna B, Radampola S (2002). Analysis of critical hydraulic gradient for particle movement in filtration. *Journal of Geotechnical and Geoenvironmental Engineering*.

[16] Qian N, Wan Z (2003). *Mechanics of Sediment Transport*.

